# Instant Coffee Is Negatively Associated with Telomere Length: Finding from Observational and Mendelian Randomization Analyses of UK Biobank

**DOI:** 10.3390/nu15061354

**Published:** 2023-03-10

**Authors:** Yudong Wei, Zengbin Li, Hao Lai, Pengyi Lu, Baoming Zhang, Lingqin Song, Lei Zhang, Mingwang Shen

**Affiliations:** 1 China-Australia Joint Research Center for Infectious Diseases, School of Public Health, Xi’an Jiaotong University Health Science Center, Xi’an 710061, China; 2 College of Stomatology, Xi’an Jiaotong University, Xi’an 710004, China; 3 Key Laboratory of Shaanxi Province for Craniofacial Precision Medicine Research, College of Stomatology, Xi’an Jiaotong University, Xi’an 710004, China; 4 School of Public Health, Xi’an Jiaotong University Health Science Center, Xi’an 710061, China; 5 Department of Oncology, The Second Affiliated Hospital, Xi’an Jiaotong University, Xi’an 710004, China; 6 Melbourne Sexual Health Centre, Alfred Health, Melbourne, VIC 3053, Australia; 7 Central Clinical School, Faculty of Medicine, Nursing and Health Sciences, Monash University, Melbourne, VIC 3800, Australia; 8 Key Laboratory for Disease Prevention and Control and Health Promotion of Shaanxi Province, Xi’an 710061, China

**Keywords:** coffee, instant coffee, telomere length, aging, Mendelian randomization

## Abstract

Telomere length, as a biomarker of accelerated aging, is closely related to many chronic diseases. We aimed to explore the association between coffee consumption and telomere length. Our study included 468,924 participants from the UK Biobank. Multivariate linear models (observational analyses) were conducted to evaluate the associations of coffee intake, instant coffee intake, and filtered coffee intake with telomere length. In addition, we evaluated the causality of these associations in Mendelian randomization (MR) analyses by four methods (inverse-variance weighted (IVW), MR pleiotropy residual sum and outlier (MR-PRESSO), MR-Egger, and weighted median). Observational analyses indicated that coffee intake and instant coffee intake were negatively correlated with telomere length, which was equal to 0.12 year of age-related decrease in telomere length for each additional cup of coffee intake (*p* < 0.001), and 0.38 year of age-related decrease in telomere length for each additional cup of instant coffee intake (*p* < 0.001), respectively. There was no significant correlation between filtered coffee and telomere length (*p* = 0.862). Mendelian randomization analyses supported the results of observational analyses. Coffee intake was found to have a causal effect on telomere length through weighted median analysis (*p* = 0.022), and instant coffee intake had a causal effect on telomere length through IVW analysis (*p* = 0.019) and MR-PRESSO analysis (*p* = 0.028). No causal relationship was found between filtered coffee intake and telomere length (*p* > 0.05). Coffee intake, particularly instant coffee, was found to have an important role in shortening telomere length.

## 1. Introduction

Telomeres, protein-protected short sequences of DNA repeats located at the ends of chromosomes, are shortened with each somatic cell cycle. Telomeres preserve hereditary information by keeping chromosomes stable, and shorten after each cell division [1,2]. Therefore, telomere length, as a biological indicator of aging, dictates the cell’s proliferative history [3]. Telomere length is linked to a variety of aging-related disorders, such as diabetes, cancers, Alzheimer’s disease, and cardiovascular disease [4,5,6,7,8]. In addition, telomere length is heritable and attributed to gender and ethnicity [9,10], and also linked to environmental and lifestyle factors such as exercise, smoking, and dietary habits [11,12,13,14]. There is a growing interest in one’s lifestyle and its potential effect on telomere length [12,15].

Coffee, as one of the most popular beverages, has been studied for its effect on health [16,17,18]. Several studies have investigated the link between coffee consumption and telomere length, but the results were controversial. A cross-sectional study of 5826 adults based on the National Health and Examination Survey (NHANES) found a positive correlation between coffee consumption and telomere length [19]. Another cross-sectional study based on 4780 women in the Nurses’ Health Study also found that coffee consumption was positively associated with telomere length [20]. A random control trial (RCT) of 37 chronic hepatitis C patients still found this positive relationship between them [21]. The other three observational studies, which respectively included 1638, 840, and 28 subjects, found no statistical association between telomere length and coffee consumption [22,23,24]. Coffee contained chlorogenic acid, caffeine, diterpenoids, and other active substances, while commercial instant coffee also contained sugar, creamer, and other flavoring agents which may account for different health effects [25,26]. Previous studies were limited by small sample sizes, and they neither classified the coffee type nor explored the causality between coffee consumption and telomere length.

Mendelian randomization (MR) is a method to explore evidence for causality [27]. MR utilizes the random distribution of genetic variants at conception to reduce and limit residual confounding and reverse causality [28]. It makes use of genetic variants as instrumental variables (IV) which are significantly related to risk factors and provides a reliable causal estimate [27,29]. MR has been used to explore the causal estimate between coffee consumption and health outcomes, such as nonalcoholic fatty liver disease [30], osteoarthritis [31], kidney stones [32], and Alzheimer’s disease [33], but the casual association between coffee consumption and telomere length was unknown [19,20,21,22,23,24]. In this paper, we aimed to investigate the relationship and causality between coffee consumption and telomere length based on UK biobank data, by using observational and MR analyses. Understanding coffee’s effect on telomere length might help discover new pathways by which coffee consumption influences health and longevity [15,26].

## 2. Methods

### 2.1. Study Population

UK biobank collected the health information of more than 500,000 participants aged 37–73 from 2006–2010. At the participants’ baseline visit, trained staff assisted in completing the touch-screen questionnaire on lifestyle factors, health-related information, and food frequency questionnaires. Meanwhile, participants accepted a comprehensive physical exam, and their biological samples were collected. The 24-h dietary recall questionnaires were collected five times online from April 2009 to June 2012. All the procedure was conducted according to the Declaration of Helsinki principles, and its protocol was reviewed and approved by the Northwest Multi-Center Research Ethics Committee (11/NW/0382). Opt-in written informed consent was obtained. The telomere length of 472,525 participants was assessed at the baseline survey. Details of the UK biobank’s explanations, design, and questionnaire are available elsewhere [34].

**Figure 1:**
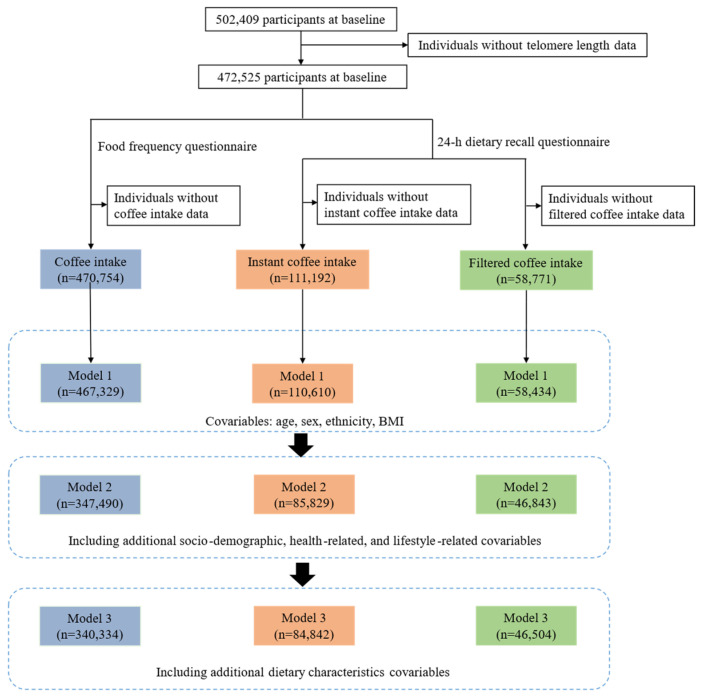
Flowchart of study participants.

There were 502,409 participants at baseline, and we included 472,525 participants with telomere length data. In the food frequency questionnaire, 470,754 participants had data on coffee intake data. After adjusting for covariables, 467,329, 347,490, and 3,403,374 participants were in model 1, model 2, and model 3, separately. In the 24-h dietary recall questionnaire, there were 111,192 individuals with instant coffee intake records and 58,771 with filtered coffee intake records. For instant coffee, 110,610, 85,829 and 84,842 participants remained in model 1, model 2, and model 3, respectively. For filtered coffee, there were 58,434, 46,843, and 46,504 participants in model 1, model 2, and model 3, respectively. The flowchart of study participants is demonstrated in Figure 1.

### 2.2. Assessment of Telomere Length

DNA was taken from peripheral blood leukocytes by the UK biobank for an array genotyping project. Research staff used multiple quantitative polymerase chain reaction (PCR) methods to measure leukocytes’ telomere length. This was represented by the ratio (T/S) of telomere repeat copy number (T) relative to a single copy gene (S). The details of operation and adjustment for technical factors are described elsewhere [35]. Our study used the Z-standardized log-telomere length measure, following the previous study [15]. In order to suggest the clinical implication of telomere length change, we transformed the 𝛽 coefficient into the equivalent years of age-related change in telomere length for each additional cup of coffee consumption. We divided the 𝛽 coefficient by 0.023, which is the 𝛽 coefficient of telomere length decreasing in age-relation per year computed in UK biobank studies [15,35].

### 2.3. Assessment of Coffee Consumption

We chose three coffee-related exposure variables from the dietary questionnaire: coffee intake, instant coffee intake, and filtered coffee intake. The coffee intake was collected from the food frequency questionnaire at baseline: “How many cups of coffee do you drink each day (including decaffeinated coffee)?”. Participants would input the number of cups, or select an option from “Less than one”, “Do not know”, or “Prefer not to answer”. Information on instant coffee intake and filtered coffee intake was gathered from the five-times questionnaires of 24-h dietary recall after they answered “Yes” in the “Did you drink any coffee yesterday”. The questions were “How many cups/mugs of instant coffee did you drink yesterday?” and “How many cups/mugs of filter/americano/cafetiere coffee did you drink yesterday?”. Participants would make the choice from the number of cups. Instant coffee and filtered coffee were counted as “6+” if the coffee consumption exceeded 6 cups. Participants were included if they completed only one of five recalls. If participants filled out the questionnaire with more than one recall, the mean intake would represent their consumption.

### 2.4. Assessment of Covariables

We included socio-demographic, health-related, lifestyle-related, and dietary characteristics as covariates for the multivariate analyses [12,15,19,20]. Socio-demographics included age, sex, ethnicity, Townsend deprivation index, and qualification. The Townsend Deprivation Index was a census-based measure of material deprivation that factored in aspects including lack of a car, overcrowded living conditions, homeownership status, and unemployment. Health-related characteristics included body-mass index (BMI), white blood cell count (WBC), C-reactive protein (CRP), vascular/heart problems diagnosed by the doctor, cancer diagnosed by the doctor, and diabetes diagnosed by the doctor. The BMI was calculated as diving weight (kg) by the square of height (m^2^), which were both measured at the baseline visit. The lifestyle-related characteristics contained total MET-minutes/week of physical activity, smoking status, and alcohol intake frequency. The metabolic equivalent task minutes per week for walking, including moderate activity and vigorous activity, were designated as a summary of the overall MET-minute/week of physical activity, which was calculated based on the International Physical Activity Questionnaire. Dietary characteristics were represented by the frequency of red-meat intake (beef, mutton, and pork), processed meat intake, oily fish intake, fruit intake (fresh and dried), and vegetable intake (cooked and salad/raw). These were all collected through a standardized and validated food frequency questionnaire (FFQ) to calculate habitual dietary intake. All measurements were gathered from the baseline survey.

### 2.5. Observational Analyses

We concluded the data of coffee intake at baseline. Besides, we clarified the coffee type into instant coffee and filtered coffee. Baseline characteristics were described by percentage (N (%)), and telomere length was calculated by the mean and standard deviation (x ± SD). One-way analysis of variance was conducted to compare the significant differences in telomere length between groups. We performed multivariate linear regression as observational analyses, and established three models to explore the relationship between coffee intake and telomere length. Model 1 included age (year; continuous), gender (male and female; categorical), ethnicity (white and others; categorical), and BMI (kg/m^2^; continuous) as covariates. Model 2 expanded on model 1 by including Townsend deprivation index (score; continuous), qualification (college or university degree, advanced/advanced subsidiary/national vocational qualification/higher national diploma/higher national certificate/other professional qualifications, ordinary levels/general certificate of secondary education/certificate of secondary education, and none of the above; categorical), WBC (10⁹ cells/L; continuous), CRP (mg/L; continuous), vascular/heart problems diagnosed by the doctor (none, heart attack, angina, stroke, and high blood pressure; categorical), cancer diagnosed by the doctor (yes, and no/don’t know; categorical), diabetes diagnosed by the doctor (yes, and no/don’t know; categorical), total MET-minutes/week physical activity (minutes/week; continuous), smoking status (current, previous and never; categorical), alcohol intake frequency (daily or almost daily, there or four times a week, once or twice a week, once to three times a month, special occasions only, and never; categorical). On the basis of model 1 and model 2, model 3 added the frequency (never, less than once a week, once a week, 2–4 times a week, and >4 times a week; categorical) of beef intake, mutton intake, pork intake, processed meat intake, and oily fish intake; and the consumption (tablespoon/day; continuous) of fresh fruit intake, dried fruit intake, cooked vegetable intake, and salad/raw vegetable intake. We separately excluded the participants in these three models if they lacked information on exposure factors and covariables, or answered the questionnaire with “Do not know” or “Prefer not to answer”. The coefficient in linear regression was tested by *t*-test.

### 2.6. Mendelian Randomization Analyses

To investigate the causal association between coffee consumption and telomere length, we conducted MR analyses using four methods: IVW, MR-PRESSO, MR-Egger, and weighted median. The IVW method is based on the assumption that there is no pleiotropy (IVs affect telomere length through alternative pathways) and that all IVs are valid [29]. The MR-Egger approach can show a valid causal effect estimate even though all IVs are invalid [36]. The weighted median approach requires that at least half of IVs are valid [29]. The MR-PRESSO approach identifies potential IV abnormalities by thoroughly testing and automatically removing identified abnormalities to provide an unbiased causal effect result [37].

In order to reduce the influence of confounding factors (including race), we chose both exposure and outcome populations from the UK biobank. Single nucleotide polymorphisms (SNPs) were extracted from the genome-wide association studies (GWAS) of the UK biobank as IVs. We selected independent SNPs (r^2^ < 0.001, window size = 10,000 kb) of coffee consumption at a level of *p* < 5 × 10^−6^. In addition, we calculated the F-score to assess the instrumental strength of SNPs. SNPs with an F score below 10 were considered weak IVs and would be removed [36]. 

We conducted the following sensitivity analyses to evaluate the robustness of MR results. (1) The fixed-effect model was performed if there was no heterogeneity in the IVW analysis; otherwise, the random-effect model was used. (2) We detected the horizontal pleiotropy by MR-Egger intercept [36] and MR-PRESSO global test [37]. (3) The leave-one-out analysis was performed to assess whether the casual effect result was driven by any single SNP. All statistical analyses were performed in R v4.1.0 (R Foundation, Vienna, Austria). “TwoSampleMR” and “MRPRESSO” packages were used for MR analyses [37,38]. *p* < 0.05 was considered to be statistically significant.

## 3. Results

### 3.1. Population Characteristics

In general, the baseline characteristics of participants in the UK Biobank are demonstrated in Table 1, in which 55.6% of the participants were younger than 60 years old, 54.2% were female, and 91.1% were white. The BMI of 67.0% of participants was over 24.9 kg/m^2^, 30.8% had more than 50 h (3000 min) of total MET physical activity per week, 22.7% of participants were at a high CRP level (>3 mg/L) [39], 29.8% of participants had vascular disease, 7.6% cancer, 5.2% diabetes, 50.5% of the participants drank more than one cup of coffee each day, and 69.1% and 55.3% of individuals who drank instant or filtered coffee yesterday consumed more than one cup.

In the univariable analysis of coffee intake, the significant differences in telomere length included age (*p* < 0.001), sex (*p* < 0.001), ethnicity (*p* < 0.001), BMI (*p* < 0.001), qualification (*p* < 0.001), Townsend deprivation index (*p* < 0.001), MET physical activity (*p* < 0.001), WBC (*p* < 0.001), CRP (*p* < 0.001), smoking status (*p* < 0.001), alcohol intake (*p* < 0.001), vascular/heart problem (*p* < 0.001), diabetes (*p* < 0.001), oily fish intake (*p* < 0.001), processed meat intake (*p* < 0.001), beef intake (*p* < 0.001), mutton/lamb intake (*p* < 0.001), pork intake (*p* < 0.001), and salad/raw vegetable intake (*p* < 0.001) (Table 1). In addition, we clarified the coffee type into instant coffee and filtered coffee in the univariable analysis. Instant coffee and filtered coffee also showed significant differences in these variables. However, cooked vegetable intake (*p* < 0.001, *p* < 0.001), fresh fruit intake (*p* < 0.001, *p* = 0.007), and coffee consumption (*p* < 0.001, *p* = 0.045) significantly differed in coffee intake and instant coffee intake. Cancer (*p* < 0.001, *p* = 0.005), and dried fruit intake (*p* < 0.001, *p* < 0.001) only showed significant differences in coffee intake and filtered coffee intake.

### 3.2. Observational Analyses of Coffee Consumption on Telomere Length

The results of the multivariable analyses of coffee consumption on telomere length are shown in Table 2. The *p*_1_, *p*_2_, and *p*_3_ represented the significance of 𝛽 coefficient in model 1, model 2, and model 3, separately. Coffee intake was inversely associated with telomere length in model 1 and was equal to 0.22 year in age-related decrease in telomere length for each additional cup of coffee intake (95% confidence interval (CI): −0.28, −0.16; *p*_1_ < 0.001). The inverse association of coffee intake with telomere length still existed in model 2 (equal to 0.13 year age-related decrease in telomere length, 95% CI: −0.20, −0.06; *p*_2_ < 0.001) and model 3 (equal to 0.12 year age-related decrease in telomere length, 95% CI: −0.19, −0.05; *p*_3_ < 0.001). Instant coffee intake had a negative correlation with telomere length in all three models, which was equal to 0.58 year age-related decrease in telomere length for each additional cup of instant coffee intake (95% CI:−0.78, −0.38; *p*_1_ < 0.001), 0.39 year age-related decrease in telomere length for each additional cup of instant coffee intake (95% CI: −0.62, −0.16; *p*_2_ < 0.001), and 0.38 year age-related decrease in telomere length for each additional cup of instant coffee intake (95% CI: −0.61, −0.15; *p*_3_ < 0.001), separately. However, the association of filtered coffee intake with telomere length was not statistically significant in the three models (*p*_1_ = 0.952, *p*_2_ = 997, *p*_3_ = 0.862).

### 3.3. MR Analyses of Coffee Consumption on Telomere Length

To check whether there was a causal connection between coffee consumption and telomere length, we further conducted two-sample MR analyses (Table 3). We identified 109, 21, and 20 SNPs for coffee intake, filtered coffee intake, and instant coffee intake, respectively. Heterogeneity was observed in the IVW analyses of coffee intake (*p* < 0.001) and filtered coffee intake (*p* = 0.006). Then the random-effect model was performed in the IVW analyses. No horizontal pleiotropy (*p* > 0.05) was found in the MR-Egger test. However, significant horizontal pleiotropy was represented in the MR-PRESSO test of coffee intake (*p* < 0.001) and filtered coffee intake (*p* = 0.010). We thus chose the corrected causal effect estimates.

The weighted median analysis suggested that coffee intake might be negatively casual associated with telomere length, equal to 3.13 (95% CI: −5.80, −0.46; *p* = 0.022) years of age-related decrease in telomere length for each additional cup of coffee intake. IVW analysis (equal to 0.85 year age-related decrease in telomere length for each additional cup of instant coffee intake, 95% CI: −1.56, −0.14; *p* = 0.019) and MR-PRESSO analysis (equal to 0.85 year age-related decrease in telomere length for each additional cup of instant coffee intake, 95% CI: −1.55, −0.15; *p* = 0.028) indicated that instant coffee intake was negatively causally associated with telomere length. No significant difference was found in any MR analysis of filtered coffee intake (*p* > 0.05).

The leave-one-out analysis suggested that the causal effect results of coffee intake and filtered coffee intake in the IVW analyses were not driven by any single SNP (Supplementary Tables S1 and S2). However, rs2472297 and rs2726351 might affect the result of instant coffee intake of IVW analysis (Supplementary Table S3), even MR-PRESSO analysis indicating that there are no outliers. Therefore, the result of instant coffee intake in IVW analysis requires careful consideration.

## 4. Discussion

In this study, we investigated the association between coffee consumption and telomere length using the data from UK biobank. We found that coffee intake and instant coffee intake were negatively correlated with telomere length, but there was no significant correlation between filtered coffee and telomere length through the observational analyses. Mendelian randomization analyses supported the results of observational analyses. We found that instant coffee had a causal relationship with telomere length in the IVW and MR-PRESSO approach, and coffee intake had a causal relationship with telomere length in the weighted median analysis. However, filtered coffee did not have a causal relationship with telomere length in the four MR analyses. To the best of our knowledge, this was the first study to evaluate the causal association between coffee consumption and telomere length in the UK biobank with a large population size. 

Previous research on the association between telomere length and coffee has not yielded a consistent result. Three studies, including a Nurses’ Health Study, an NHANES survey, and a randomized controlled trial of chronic hepatitis C patients, revealed that higher coffee consumption was associated with longer telomere length [19,20,21]. Other epidemiological studies indicated that there was no association between coffee consumption and telomere length [22,23,24]. Our results indicated that coffee intake was negatively correlated with telomere length when the coffee subtype was not considered. After exploring the relationship by subtype, instant coffee intake showed a negative association with telomere length, while filtered coffee intake did not. More than half of the UK biobank participants preferred drinking instant coffee [40]. We inferred that instant coffee played a key role in the association that coffee consumption had a negative effect on telomere length. Previous studies did not take coffee types into account, and the inconsistent results might be attributed to small sample sizes or a lack of a classification of coffee type.

Instant coffee might shorten telomere length, and it might lead to the occurrence and development of diseases. The health effects of instant coffee, which varied from other subtypes of coffee, might be caused by their different ingredients. The mineral lead in instant coffee was more abundant than that in other coffee types, and long-term consumption of instant coffee may result in excessive lead [41]. Additional substances added to commercial instant coffee, such as creamer and flavoring agents, might partially explain the negative effect [25,26]. Some studies have investigated the effects of coffee subtypes on health. Ground coffee could reduce the risk of type 2 diabetes, whereas instant coffee might increase the risk [42,43]. Instant coffee consumption has been proven to be associated with obesity [44,45]. Compared to women who did not regularly drink coffee, those who drank instant coffee had a higher risk of developing breast cancer [46]. Instant coffee was regarded as a risk factor for Alzheimer’s disease and frailty in the elderly [47,48]. Instant coffee might have the effect of shortening telomere length, and might lead to the occurrence and development of diseases. Therefore, we emphasized the importance of coffee types and the consumption of instant coffee at an appropriate amount. More research needs to identify whether the ingredients in instant coffee results in shorter telomere length.

Our study has several limitations. First, coffee consumption was assessed by frequency questionnaire, while instant coffee intake and filtered coffee intake were based on 24-h recall questionnaires, which might have led to biases in the results. Second, in the 24-h recall questionnaire, the participants who consumed more than six cups were recorded as “6+”, which might weaken the association because of information loss. Third, we didn’t further classify coffee types with milk. Coffee with milk (including expresso, cappuccino, and latte) might have effects on telomere length. Fourth, we relaxed the *p*-value threshold (*p* < 5 × 10^−6^) of SNPs due to lack of sufficient SNPs (less than three) after linkage disequilibrium, resulting in the reduced robustness of MR results. Fifth, in the observational analyses, although exhaustive adjustment was conducted in the multivariable analyses, residuals or unmeasured confounders could not be excluded [49,50,51]. Finally, our analyses were limited to individuals of European ancestry. Generalization to other ethnic or regional populations requires careful consideration.

## 5. Conclusions

In summary, we found that both observational and Mendelian randomization analyses indicated that coffee intake, especially instant coffee, might reduce telomere length, while filtered coffee did not. The type of coffee plays a key role in the effect of coffee consumption on telomere length. Further studies are needed to validate our findings and clarify the potential mechanism by which coffee consumption is associated with telomere length.

## Figures and Tables

**Table 1:** Baseline characteristics by coffee consumption in the UK Biobank.

Exposure	Coffee Intake			Instant Coffee Intake			Filtered Coffee Intake		
	N (%)	Telomere Length (z-Score), x ± SD	*p*	N (%)	Telomere Length (z-score), x ± SD	*p*	N (%)	Telomere Length (z-Score), x ± SD	*p*
Covariables included in model 1									
Age			<0.001			<0.001			<0.001
<50	110,071 (23.4)	0.27 ± 0.98		24,273 (21.8)	0.27 ± 0.97		12,461 (21.2)	0.31 ± 0.98	
50–59	156,395 (33.2)	0.06 ± 0.98		38,181 (34.3)	0.07 ± 0.97		20,843 (35.5)	0.11 ± 0.97	
60–69	202,027 (42.9)	−0.19 ± 0.98		48,264 (43.4)	−0.16 ± 0.98		25,255 (43.0)	−0.12 ± 0.98	
>69	2261 (0.5)	−0.35 ± 0.97		474 (0.4)	−0.32 ± 0.94		212 (0.4)	−0.33 ± 0.98	
Sex			<0.001			<0.001			<0.001
female	255,249 (54.2)	0.09 ± 1.00		59,742 (53.7)	0.11 ± 0.99		31,565 (53.7)	0.15 ± 0.98	
male	215,505 (45.8)	−0.10 ± 1.00		51,450 (46.3)	−0.09 ± 0.99		27,206 (46.3)	−0.06 ± 0.99	
Ethnicity			<0.001			<0.001			<0.001
white	427,173 (91.1)	−0.02 ± 1.00		103,351 (93.2)	0.00 ± 0.99		53,217 (90.9)	0.04 ± 0.99	
others	41,974 (8.9)	0.18 ± 1.02		7490 (6.8)	0.18 ± 1.00		5332 (9.1)	0.17 ± 0.97	
Body mass index, kg/m^2^			<0.001			<0.001			<0.001
<18.5	2407 (0.5)	0.13 ± 1.05		530 (0.5)	0.17 ± 0.95		336 (0.6)	0.19 ± 0.94	
18.5–24.9	152,556 (32.5)	0.07 ± 1.00		38,452 (34.7)	0.07 ± 0.99		24,126 (41.1)	0.11 ± 0.98	
25–29.9	199,580 (42.6)	−0.02 ± 1.00		47,370 (42.7)	−0.01 ± 0.99		24,157 (41.2)	0.02 ± 0.99	
>29.9	114,381 (24.4)	−0.05 ± 1.00		24,607 (22.2)	−0.03 ± 0.99		10,037 (17.1)	−0.01 ± 1.00	
Additional covariables included in model 2									
Qualification			<0.001			<0.001			<0.001
college or university degree	152,641 (32.8)	0.09 ± 1.00		43,330 (39.1)	0.07 ± 0.99		34,410 (58.7)	0.08 ± 0.98	
A/AS/NVQ/HND/HNC/other professional qualifications	107,766 (23.1)	−0.01 ± 1.00		27,267 (24.6)	−0.01 ± 0.99		12,248 (20.9)	0.03 ± 1.00	
O levels/GCSEs/CSEs	125,148 (26.9)	0.01 ± 0.99		12,248 (20.9)	0.03 ± 1.00		9836 (16.8)	0.02 ± 0.99	
none of the above	80,245 (17.2)	−0.17 ± 0.99		10,310 (9.3)	−0.14 ± 1.00		2117 (3.6)	−0.14 ± 1.02	
Townsend deprivation index (percentile)			<0.001			<0.001			<0.001
<25	117,494 (25.0)	−0.01 ± 0.99		27,758 (25.0)	0.00 ± 0.99		27,758 (25.0)	0.00 ± 0.99	
25–49	117,591 (25.0)	−0.01 ± 1.00		27,769 (25.0)	0.00 ± 0.99		27,769 (25.0)	0.00 ± 0.99	
50–75	117,545 (25.0)	0.01 ± 1.00		27,768 (25.0)	0.02 ± 0.99		27,768 (25.0)	0.02 ± 0.99	
>75	117,545 (25.0)	0.02 ± 1.01		27,765 (25.0)	0.04 ± 1.00		27,765 (25.0)	0.04 ± 1.00	
Total MET physical activity, minutes/week			<0.001			<0.001			0.091
<600	71,452 (18.7)	0.00 ± 1.00		17,394 (18.6)	0.02 ± 0.99		8619 (16.9)	0.06 ± 0.99	
600–3000	192,526 (50.5)	0.02 ± 1.00		49,514 (52.8)	0.03 ± 0.99		28,805 (56.3)	0.06 ± 0.99	
>3000	117,373 (30.8)	−0.01 ± 1.00		26,841 (28.6)	−0.01 ± 1.00		13,696 (26.8)	0.04 ± 0.98	
White blood cell count, 10⁹ cells/L (percentile)			<0.001			<0.001			<0.001
<5	21,096 (4.6)	0.16 ± 1.01		5204 (4.8)	0.14 ± 0.99		2814 (4.9)	0.17 ± 0.97	
5–24	92,706 (20.3)	0.07 ± 0.99		21,087 (19.5)	0.08 ± 0.99		11,363 (19.9)	0.1 ± 0.99	
25–74	228,615 (50.0)	−0.01 ± 0.99		54,609 (50.6)	0.01 ± 0.98		28,491 (49.9)	0.04 ± 0.98	
75–95	91,658 (20.1)	−0.06 ± 1.01		21,585 (20.0)	−0.04 ± 1.01		11,511 (20.2)	0.00 ± 0.99	
>95	22,868 (5.0)	−0.08 ± 1.04		5419 (5.0)	−0.05 ± 1.01		2911 (5.1)	0.02 ± 1.04	
C-reactive protein, mg/L			<0.001			<0.001			<0.001
<1	175,766 (39.2)	0.06 ± 1.00		44,634 (42.2)	0.07 ± 0.99		27,497 (49.2)	0.10 ± 0.98	
1–3	170,810 (38.1)	−0.03 ± 1.00		39,786 (37.6)	−0.02 ± 0.99		19,608 (35.1)	0.01 ± 0.99	
>3	101,649 (22.7)	−0.06 ± 1.01		21,298 (20.1)	−0.02 ± 0.99		8789 (15.7)	−0.01 ± 1.01	
Smoking status			<0.001			<0.001			<0.001
current	49,564 (10.6)	−0.06 ± 1.01		9422 (8.5)	−0.02 ± 1.01		3950 (6.7)	0.02 ± 1.01	
previous	162,837 (34.7)	−0.06 ± 1.00		40,079 (36.1)	−0.04 ± 0.99		32,949 (56.2)	0.09 ± 0.99	
never	256,600 (54.7)	0.05 ± 1.00		61,411 (55.4)	0.06 ± 0.99		32,949 (56.2)	0.09 ± 0.99	
Alcohol intake frequency			<0.001			<0.001			<0.001
daily or almost daily	95,928 (20.4)	−0.06 ± 1.00		25,082 (22.6)	−0.04 ± 0.99		17,836 (30.4)	0.00 ± 0.99	
three or four times a week	108,846 (23.1)	0.00 ± 0.99		28,790 (25.9)	0.01 ± 0.99		17,558 (29.9)	0.06 ± 0.98	
once or twice a week	121,492 (25.8)	0.01 ± 1.00		28,810 (25.9)	0.04 ± 0.99		13,149 (22.4)	0.09 ± 0.99	
once to three times a month	52,410 (11.1)	0.04 ± 0.99		12,318 (11.1)	0.05 ± 0.99		4853 (8.3)	0.12 ± 0.99	
special occasions only	54,010 (11.5)	0.03 ± 1.01		10,465 (9.4)	0.06 ± 1.01		3223 (5.5)	0.08 ± 1.00	
never	37,680 (8.0)	0.02 ± 1.02		5658 (5.1)	0.01 ± 1.01		2110 (3.6)	0.04 ± 1.02	
Vascular/heart problems diagnosed by doctor			<0.001			<0.001			<0.001
none	329,721 (70.2)	0.04 ± 1.00		80,577 (72.6)	0.05 ± 0.99		44,727 (76.2)	0.08 ± 0.99	
heart attack	10,866 (2.3)	−0.29 ± 1.00		2048 (1.8)	−0.31 ± 1.02		741 (1.3)	−0.26 ± 1.01	
angina	10,604 (2.3)	−0.25 ± 0.99		1952 (1.8)	−0.25 ± 0.99		744 (1.3)	−0.19 ± 0.94	
stroke	5768 (1.2)	−0.20 ± 1.02		1073 (1.0)	−0.21 ± 1.03		463 (0.8)	−0.12 ± 1.03	
high blood pressure	112,761 (24.0)	−0.06 ± 0.99		25,384 (22.9)	−0.05 ± 0.99		12,022 (20.5)	−0.02 ± 0.99	
Cancer diagnosed by doctor			<0.001			0.082			0.005
yes	35,848 (7.6)	−0.05 ± 1.01		8386 (7.5)	0.02 ± 0.99		4448 (7.6)	0.01 ± 0.98	
no/don’t know	434,636 (92.4)	0.00 ± 1.00		102,752 (92.5)	−0.03 ± 1.01		54,290 (92.4)	0.06 ± 0.99	
Diabetes diagnosed by doctor			<0.001			<0.001			<0.001
yes	24,601 (5.2)	−0.16 ± 1.01		4885 (4.4)	−0.13 ± 1.00		1922 (3.3)	−0.15 ± 1.00	
no/don’t know	445,921 (94.8)	0.01 ± 1.00		106,263 (95.6)	0.02 ± 0.99		56,820 (96.7)	0.06 ± 0.99	
Additional covariables included in model 3									
Oily fish intake			<0.001			0.027			<0.001
never	51,360 (11.0)	0.00 ± 1.00		9961 (9.0)	0.03 ± 0.98		3887 (6.6)	0.10 ± 0.98	
less than once a week	155,199 (33.2)	0.01 ± 1.00		38,576 (34.8)	0.03 ± 0.99		18,613 (31.7)	0.07 ± 0.99	
once a week	177,076 (37.8)	−0.01 ± 1.00		43,470 (39.2)	0.00 ± 1.00		24,699 (42.1)	0.04 ± 1.00	
2–4 times a week	79,993 (17.1)	−0.01 ± 1.00		18,042 (16.3)	0.00 ± 0.99		10,988 (18.7)	0.03 ± 0.98	
>4 times a week	4454 (1.0)	0.03 ± 1.01		865 (0.8)	0.00 ± 0.98		498 (0.8)	0.01 ± 1.04	
Processed meat intake			<0.001			<0.001			<0.001
never	43,644 (9.3)	0.09 ± 1.00		8896 (8.0)	0.13 ± 1.00		8896 (8.0)	0.13 ± 1.00	
less than once a week	142,958 (30.4)	0.03 ± 1.00		34,836 (31.4)	0.03 ± 0.99		34,836 (31.4)	0.03 ± 0.99	
once a week	137,232 (29.2)	−0.02 ± 1.00		32,351 (29.1)	0.01 ± 1.00		32,351 (29.1)	0.01 ± 1.00	
2–4 times a week	127,300 (27.1)	−0.04 ± 1.00		30,614 (27.6)	−0.02 ± 0.99		30,614 (27.6)	−0.02 ± 0.99	
>4 times a week	18,634 (4.0)	−0.05 ± 1.00		4394 (4.0)	−0.02 ± 0.99		4394 (4.0)	−0.02 ± 0.99	
Beef intake			<0.001			<0.001			<0.001
never	51,841 (11.1)	0.09 ± 1.00		10,305 (9.3)	0.12 ± 0.99		6939 (11.8)	0.15 ± 0.97	
less than once a week	213,574 (45.6)	0.00 ± 1.00		53,884 (48.6)	0.01 ± 0.99		28,430 (48.4)	0.05 ± 0.99	
once a week	149,237 (31.8)	−0.02 ± 1.00		35,002 (31.5)	0.01 ± 0.99		17,738 (30.2)	0.04 ± 0.98	
2–4 times a week	52,956 (11.3)	−0.03 ± 1.01		11,630 (10.5)	−0.01 ± 1.01		5543 (9.4)	0.03 ± 1.01	
>4 times a week	1192 (0.3)	0.06 ± 1.07		140 (0.1)	0.07 ± 0.99		38 (0.1)	0.12 ± 1.09	
Mutton/lamb intake ^+^			<0.001			<0.001			<0.001
never	83,190 (17.8)	0.05 ± 1.00		16,912 (15.3)	0.08 ± 0.98		8839 (15.1)	0.13 ± 0.97	
less than once a week	265,065 (56.7)	0.00 ± 1.00		67,310 (60.7)	0.02 ± 0.99		34,599 (59.0)	0.05 ± 0.99	
once a week	105,231 (22.5)	−0.04 ± 1.00		23,934 (21.6)	−0.02 ± 0.99		13,686 (23.3)	0.01 ± 1.00	
2–4 times a week	13,739 (2.9)	−0.01 ± 1.02		2652 (2.4)	−0.03 ± 1.01		1527 (2.6)	−0.01 ± 1.04	
>4 times a week	500 (0.1)	0.16 ± 1.02		33 (0.0)	0.31 ± 0.84		3 (0.0)	0.83 ± 0.79	
Pork intake			<0.001			<0.001			<0.001
never	80,560 (17.2)	0.07 ± 1.00		15,136 (13.7)	0.09 ± 0.99		8982 (15.3)	0.14 ± 0.98	
less than once a week	266,181 (56.9)	0.00 ± 1.00		66,580 (60.0)	0.01 ± 0.99		35,572 (60.6)	0.04 ± 0.99	
once a week	104,452 (22.3)	−0.04 ± 1.00		25,448 (23.0)	−0.02 ± 0.99		12,391 (21.1)	0.02 ± 0.99	
2–4 times a week	16,058 (3.4)	0.00 ± 1.01		3629 (3.3)	−0.02 ± 1.00		1678 (2.9)	0.04 ± 0.99	
>4 times a week	657 (0.1)	0.09 ± 0.95		91 (0.1)	−0.01 ± 0.92		32 (0.1)	0.21 ± 0.89	
Cooked vegetable intake, tablespoons/day			<0.001			<0.001			0.248
0	13,909 (3.0)	−0.02 ± 1.01		2375 (2.1)	0.02 ± 1.00		766 (1.3)	0.03 ± 1.02	
<2	71,064 (15.3)	0.03 ± 1.00		16,280 (14.7)	0.04 ± 0.98		7677 (13.1)	0.07 ± 0.98	
2–3	281,185 (60.5)	0.00 ± 1.00		69,530 (62.9)	0.01 ± 0.99		37,264 (63.6)	0.05 ± 1.00	
4–5	75,667 (16.3)	−0.02 ± 1.00		17,403 (15.7)	−0.01 ± 1.00		10,059 (17.2)	0.04 ± 0.98	
6+	23,156 (5.0)	0.00 ± 1.01		4913 (4.4)	0.02 ± 0.99		2812 (4.8)	0.06 ± 0.99	
Salad/raw vegetable intake, tablespoons/day			<0.001			<0.001			0.001
0	45,332 (9.8)	−0.06 ± 1.01		9183 (8.3)	−0.03 ± 1		3076 (5.3)	0.00 ± 1.00	
(0, 1]	161,209 (34.7)	0.00 ± 1.00		40,654 (36.8)	0.01 ± 0.99		20,377 (34.8)	0.05 ± 0.98	
(1, 3]	178,476 (38.4)	0.00 ± 0.99		42,626 (38.6)	0.02 ± 0.98		24,330 (41.6)	0.05 ± 0.99	
(3, 5]	56,054 (12.1)	0.02 ± 1.01		12,716 (11.5)	0.04 ± 1.01		7446 (12.7)	0.07 ± 1.00	
>5	23,612 (5.1)	0.06 ± 1.00		5215 (4.7)	0.05 ± 1		3301 (5.6)	0.10 ± 0.98	
Fresh fruit intake, tablespoons/day			<0.001			0.007			0.215
0	28,801 (6.1)	−0.04 ± 1.02		5517 (5.0)	−0.01 ± 0.99		1836 (3.1)	0.03 ± 0.99	
(0, 1]	137,737 (29.4)	−0.01 ± 1.00		32,592 (29.4)	0.00 ± 0.99		15,904 (27.1)	0.05 ± 0.99	
(1, 3]	226,434 (48.3)	0.01 ± 1.00		55,815 (50.3)	0.02 ± 0.99		30,665 (52.2)	0.05 ± 0.99	
(3, 5]	63,066 (13.5)	0.02 ± 0.99		14,417 (13.0)	0.03 ± 1.00		8658 (14.8)	0.07 ± 1.00	
>5	12,834 (2.7)	0.01 ± 1.00		2642 (2.4)	0.01 ± 1.00		1632 (2.8)	0.04 ± 0.97	
Dried fruit intake, tablespoons/day			<0.001			0.690			<0.001
0	251,959 (54.1)	−0.02 ± 1.00		56,075 (50.8)	0.00 ± 0.99		23,912 (40.9)	0.03 ± 0.99	
(0, 1]	134,229 (28.8)	0.03 ± 0.99		35,389 (32.1)	0.04 ± 0.99		21,833 (37.4)	0.07 ± 0.98	
(1, 3]	54,085 (11.6)	0.02 ± 1.00		13,063 (11.8)	0.02 ± 1.00		8737 (15.0)	0.06 ± 0.99	
(3, 5]	15,951 (3.4)	0.01 ± 1.01		3723 (3.4)	0.00 ± 1.00		2607 (4.5)	0.08 ± 0.99	
>5	9281 (2.0)	0.00 ± 1.01		2163 (2.0)	0.02 ± 0.99		1350 (2.3)	0.06 ± 1.02	
Coffee consumption, cup			<0.001			0.045			0.490
(0, 1]	232,910 (49.5)	0.02 ± 1.00		34,325 (30.9)	0.03 ± 0.99		26,280 (44.7)	0.05 ± 0.99	
(1, 3]	145,997 (31.0)	0.00 ± 1.00		56,310 (50.6)	0.01 ± 0.99		29,079 (49.5)	0.06 ± 0.99	
3+	91,847 (19.5)	−0.04 ± 1.00		20,557 (18.5)	0.00 ± 1.00		3412 (5.8)	0.04 ± 1.00	

+: 2–4 times a week and >4 times a week were combined in the filtered coffee analysis. Abbreviations: A, Advanced; AS, Advanced Subsidiary; CSE, Certificate of Secondary Education; GCSE, General Certificate of Secondary Education; O, Ordinary; HNC, Higher National Certificate; HND, Higher National Diploma; NVQ, National Vocational Qualification; SD, standard deviation.

**Table 2:** Association of coffee consumption and telomere length in multivariable linear regression.

Exposure	Model 1 ^1^			Model 2 ^2^			Model 3 ^3^		
	n	Effect in Years (95% CI) ^4^	*p*	n	Effect in Years (95% CI) ^4^	*p*	n	Effect in Years (95% CI) ^4^	*p*
Coffee intake	467,329	−0.22 (−0.28, −0.16)	<0.001	347,490	−0.13 (−0.20, −0.06)	<0.001	340,334	−0.12 (−0.19, −0.05)	<0.001
Instant coffee intake	110,610	−0.58 (−0.78, −0.38)	<0.001	85,829	−0.39 (−0.62, −0.16)	<0.001	84,842	−0.38 (−0.61, −0.15)	0.001
Filtered coffee intake	58,434	0.01 (−0.37, 0.40)	0.952	46,843	0.01 (−0.42, 0.44)	0.997	46,504	−0.04 (−0.47, 0.39)	0.862

^1^ Covariables in model 1: age, sex, ethnicity, BMI. ^2^ Covariables in model 2: Model 1 + Townsend deprivation index, total MET, WBC, CRP, qualification, smoking status, alcohol intake frequency, vascular/heart problems diagnosed by doctor, cancer diagnosed by doctor, diabetes diagnosed by doctor. ^3^ Covariables in model 3: Model 2 + oily fish intake, processed meat intake, beef intake, mutton/lamb intake, pork intake, cooked vegetable intake, salad/raw vegetable intake, fresh fruit intake, dried fruit intake. ^4^ Effect in years represents the year of age-related change in telomere length for each additional cup of coffee consumption. It was calculated by dividing *β* coefficient of multiple linear regression by the *β* coefficient of age-related telomere length decrease (0.023 per year).

**Table 3:** Causal effects of coffee consumption on telomere length estimated by Mendelian randomization.

Exposure	nSNP	IVW		MR-PRESSO		MR Egger		Weighted Median		Pleiotropy *p*		Heterogeneity *p*
		Effect in Years ^1^ (95% CI)	*p*	Effect in Years ^1^ (95% CI)	*p*	Effect in Years ^1^ (95% CI)	*p*	Effect in Years ^1^ (95% CI)	*p*	MR-PRESSO	MR Egger	
Coffee intake	109	−1.03 (−4.23, 2.18)	0.529	−1.45 (−3.53, 0.62)	0.172	−4.55 (−11.40, 2.29)	0.195	−3.13 (−5.80, −0.46)	0.022	<0.001	0.155	<0.001
Instant coffee intake	20	−0.85 (−1.56, −0.14)	0.019	−0.85 (−1.55, −0.15)	0.028	−1.31 (−3.07, 0.45)	0.162	−0.47 (−1.53, 0.58)	0.377	0.481	0.965	0.497
Filtered coffee intake	21	−0.22 (−1.77, 1.33)	0.782	−0.61 (−2.01, 0.80)	0.409	1.89 (−3.16, 6.94)	0.473	0.59 (−1.12, 2.30)	0.498	0.010	0.070	0.006

^1^ Effect in years represents the year of age-related change in telomere length for each additional cup of coffee consumption. It was calculated by dividing *β* coefficient of Mendelian randomization by the *β* coefficient of age-related telomere length decrease (0.023 per year). Abbreviations: IVW, inverse-variance weighted; MR, Mendelian randomization; MR-PRESSO, Mendelian randomization pleiotropy residual sum and outlier; SNP, Single nucleotide polymorphisms.

## Data Availability

The data of UK Biobank are available in a public, open access repository. This research has been conducted using the UK Biobank Resource under application number 79244. The UK Biobank data are available on application to the UK Biobank (www.ukbiobank.ac.uk/, accessed on 17 August 2022).

## Supplementary Materials

The following supporting information can be downloaded at: https://www.mdpi.com/article/10.3390/nu15061354/s1, Table S1: The leave-one-out analysis of coffee intake on telomere length using IVW approach; Table S2: The leave-one-out analysis of filtered coffee intake on telomere length using IVW approach; Table S3: The leave-one-out analysis of instant coffee intake on telomere length using IVW approach.
