# Supplementary material for: Instant Coffee Is Negatively Associated with Telomere Length: Finding from Observational and Mendelian Randomization Analyses of UK Biobank
Source: Nutrients. 2023 Mar 10;15(6):1354. doi: 10.3390/nu15061354 (PMC10055626; doi:10.3390/nu15061354)
Supplement: Supplementary file 1 [file nutrients-15-01354-s001.zip › nutrients-2228600-supplementary.pdf]

Contents

Supplementary Table S1. The leave-one-out analysis of coffee intake on telomere length using IVW approach.

Supplementary Table S2. The leave-one-out analysis of filtered coffee intake on telomere length using IVW approach.

Supplementary Table S3. The leave-one-out analysis of instant coffee intake on telomere length using IVW approach.

Supplementary Table S1. The leave-one-out analysis of coffee intake on telomere length using IVW approach.

Exposure	Outcome	GWAS ID of exposure	GWAS ID of outcome	Sample size	SNP	*β*	se	*p*
Coffee intake	telomere length	ukb-b-5237	ieu-b-4879	472174	rs10053913	-0.02387	0.037899	0.528737
Coffee intake	telomere length	ukb-b-5237	ieu-b-4879	472174	rs10193706	-0.02242	0.03787	0.553889
Coffee intake	telomere length	ukb-b-5237	ieu-b-4879	472174	rs10500356	-0.02254	0.037867	0.55165
Coffee intake	telomere length	ukb-b-5237	ieu-b-4879	472174	rs1057868	-0.03433	0.037835	0.364252
Coffee intake	telomere length	ukb-b-5237	ieu-b-4879	472174	rs10938398	-0.02436	0.037873	0.52004
Coffee intake	telomere length	ukb-b-5237	ieu-b-4879	472174	rs10992783	-0.01977	0.037499	0.598092
Coffee intake	telomere length	ukb-b-5237	ieu-b-4879	472174	rs11032480	-0.022	0.037829	0.560812
Coffee intake	telomere length	ukb-b-5237	ieu-b-4879	472174	rs11236225	-0.0222	0.037858	0.55762
Coffee intake	telomere length	ukb-b-5237	ieu-b-4879	472174	rs11678584	-0.02351	0.037918	0.535213
Coffee intake	telomere length	ukb-b-5237	ieu-b-4879	472174	rs116814395	-0.02461	0.037859	0.515708
Coffee intake	telomere length	ukb-b-5237	ieu-b-4879	472174	rs117514553	-0.0238	0.037887	0.52986
Coffee intake	telomere length	ukb-b-5237	ieu-b-4879	472174	rs117810762	-0.02337	0.037934	0.537866
Coffee intake	telomere length	ukb-b-5237	ieu-b-4879	472174	rs117968677	-0.02526	0.037879	0.504802
Coffee intake	telomere length	ukb-b-5237	ieu-b-4879	472174	rs1182580	-0.02326	0.037895	0.539292

Exposure	Outcome	GWAS ID of exposure	GWAS ID of outcome	Sample size	SNP	*β*	se	*p*
Coffee intake	telomere length	ukb-b-5237	ieu-b-4879	472174	rs12514566	-0.0265	0.037889	0.484369
Coffee intake	telomere length	ukb-b-5237	ieu-b-4879	472174	rs12604773	-0.02756	0.037571	0.463226
Coffee intake	telomere length	ukb-b-5237	ieu-b-4879	472174	rs12649199	-0.02362	0.03789	0.533072
Coffee intake	telomere length	ukb-b-5237	ieu-b-4879	472174	rs12930957	-0.02326	0.037895	0.539349
Coffee intake	telomere length	ukb-b-5237	ieu-b-4879	472174	rs12989746	-0.02407	0.037926	0.525623
Coffee intake	telomere length	ukb-b-5237	ieu-b-4879	472174	rs13054099	-0.03114	0.037018	0.400219
Coffee intake	telomere length	ukb-b-5237	ieu-b-4879	472174	rs13096450	-0.02304	0.037887	0.543124
Coffee intake	telomere length	ukb-b-5237	ieu-b-4879	472174	rs13163336	-0.02298	0.037993	0.545282
Coffee intake	telomere length	ukb-b-5237	ieu-b-4879	472174	rs13378240	-0.02118	0.03776	0.574894
Coffee intake	telomere length	ukb-b-5237	ieu-b-4879	472174	rs13378244	-0.02435	0.037883	0.520306
Coffee intake	telomere length	ukb-b-5237	ieu-b-4879	472174	rs1338549	-0.02468	0.037923	0.515132
Coffee intake	telomere length	ukb-b-5237	ieu-b-4879	472174	rs13387939	-0.02237	0.038036	0.556512
Coffee intake	telomere length	ukb-b-5237	ieu-b-4879	472174	rs13437947	-0.0241	0.037884	0.52467
Coffee intake	telomere length	ukb-b-5237	ieu-b-4879	472174	rs1421085	-0.01712	0.038152	0.653532
Coffee intake	telomere length	ukb-b-5237	ieu-b-4879	472174	rs144620429	-0.02547	0.037805	0.500548

Exposure	Outcome	GWAS ID of exposure	GWAS ID of outcome	Sample size	SNP	*β*	se	*p*
Coffee intake	telomere length	ukb-b-5237	ieu-b-4879	472174	rs1447365	-0.02411	0.037891	0.524627
Coffee intake	telomere length	ukb-b-5237	ieu-b-4879	472174	rs1527961	-0.02562	0.037869	0.498706
Coffee intake	telomere length	ukb-b-5237	ieu-b-4879	472174	rs16930598	-0.02294	0.037883	0.544751
Coffee intake	telomere length	ukb-b-5237	ieu-b-4879	472174	rs17144566	-0.02223	0.037884	0.557347
Coffee intake	telomere length	ukb-b-5237	ieu-b-4879	472174	rs17842490	-0.016	0.037392	0.66878
Coffee intake	telomere length	ukb-b-5237	ieu-b-4879	472174	rs1942965	-0.02268	0.037907	0.549724
Coffee intake	telomere length	ukb-b-5237	ieu-b-4879	472174	rs2126069	-0.02338	0.037921	0.537575
Coffee intake	telomere length	ukb-b-5237	ieu-b-4879	472174	rs2189234	-0.02262	0.037935	0.551003
Coffee intake	telomere length	ukb-b-5237	ieu-b-4879	472174	rs2231142	-0.02867	0.037319	0.442265
Coffee intake	telomere length	ukb-b-5237	ieu-b-4879	472174	rs2350804	-0.02264	0.037874	0.549908
Coffee intake	telomere length	ukb-b-5237	ieu-b-4879	472174	rs2373834	-0.0242	0.037878	0.522904
Coffee intake	telomere length	ukb-b-5237	ieu-b-4879	472174	rs2400677	-0.02555	0.037828	0.49944
Coffee intake	telomere length	ukb-b-5237	ieu-b-4879	472174	rs2465037	-0.02325	0.037957	0.540117
Coffee intake	telomere length	ukb-b-5237	ieu-b-4879	472174	rs2472297	-0.0061	0.040439	0.880191
Coffee intake	telomere length	ukb-b-5237	ieu-b-4879	472174	rs2521501	-0.02294	0.037907	0.544999

Exposure	Outcome	GWAS ID of exposure	GWAS ID of outcome	Sample size	SNP	***β***	se	***p***
Coffee intake	telomere length	ukb-b-5237	ieu-b-4879	472174	rs2577374	-0.02331	0.037912	0.538611
Coffee intake	telomere length	ukb-b-5237	ieu-b-4879	472174	rs2595223	-0.02703	0.037638	0.472674
Coffee intake	telomere length	ukb-b-5237	ieu-b-4879	472174	rs2597805	-0.02445	0.03792	0.519067
Coffee intake	telomere length	ukb-b-5237	ieu-b-4879	472174	rs2842183	-0.02703	0.037639	0.472624
Coffee intake	telomere length	ukb-b-5237	ieu-b-4879	472174	rs28490963	-0.02312	0.037892	0.541683
Coffee intake	telomere length	ukb-b-5237	ieu-b-4879	472174	rs329122	-0.01688	0.03703	0.648574
Coffee intake	telomere length	ukb-b-5237	ieu-b-4879	472174	rs34060476	-0.02049	0.037961	0.589362
Coffee intake	telomere length	ukb-b-5237	ieu-b-4879	472174	rs34276507	-0.02497	0.037841	0.509311
Coffee intake	telomere length	ukb-b-5237	ieu-b-4879	472174	rs35303290	-0.02207	0.037853	0.559863
Coffee intake	telomere length	ukb-b-5237	ieu-b-4879	472174	rs4410790	-0.02887	0.040321	0.474023
Coffee intake	telomere length	ukb-b-5237	ieu-b-4879	472174	rs442355	-0.02456	0.037939	0.517415
Coffee intake	telomere length	ukb-b-5237	ieu-b-4879	472174	rs4615895	-0.02239	0.037961	0.555321
Coffee intake	telomere length	ukb-b-5237	ieu-b-4879	472174	rs476828	-0.02233	0.038149	0.558269
Coffee intake	telomere length	ukb-b-5237	ieu-b-4879	472174	rs4836963	-0.02445	0.037873	0.518573
Coffee intake	telomere length	ukb-b-5237	ieu-b-4879	472174	rs4840338	-0.02451	0.037869	0.517413

Exposure	Outcome	GWAS ID of exposure	GWAS ID of outcome	Sample size	SNP	*β*	se	*p*
Coffee intake	telomere length	ukb-b-5237	ieu-b-4879	472174	rs4937720	-0.02132	0.037743	0.572145
Coffee intake	telomere length	ukb-b-5237	ieu-b-4879	472174	rs4938000	-0.02528	0.037835	0.503955
Coffee intake	telomere length	ukb-b-5237	ieu-b-4879	472174	rs4970968	-0.02325	0.03789	0.539533
Coffee intake	telomere length	ukb-b-5237	ieu-b-4879	472174	rs4984636	-0.02436	0.037876	0.520042
Coffee intake	telomere length	ukb-b-5237	ieu-b-4879	472174	rs516636	-0.02181	0.037888	0.56493
Coffee intake	telomere length	ukb-b-5237	ieu-b-4879	472174	rs55716899	-0.02464	0.037872	0.51536
Coffee intake	telomere length	ukb-b-5237	ieu-b-4879	472174	rs55780162	-0.02335	0.037889	0.537762
Coffee intake	telomere length	ukb-b-5237	ieu-b-4879	472174	rs55895231	-0.02404	0.037882	0.525634
Coffee intake	telomere length	ukb-b-5237	ieu-b-4879	472174	rs56113850	-0.02765	0.037909	0.465827
Coffee intake	telomere length	ukb-b-5237	ieu-b-4879	472174	rs56212739	-0.02082	0.03771	0.580875
Coffee intake	telomere length	ukb-b-5237	ieu-b-4879	472174	rs56349356	-0.02304	0.037873	0.542939
Coffee intake	telomere length	ukb-b-5237	ieu-b-4879	472174	rs56791931	-0.0231	0.037892	0.542172
Coffee intake	telomere length	ukb-b-5237	ieu-b-4879	472174	rs6030315	-0.0426	0.027412	0.120185
Coffee intake	telomere length	ukb-b-5237	ieu-b-4879	472174	rs6062682	-0.02306	0.037962	0.543542
Coffee intake	telomere length	ukb-b-5237	ieu-b-4879	472174	rs6063085	-0.02017	0.037784	0.59354

Exposure	Outcome	GWAS ID of exposure	GWAS ID of outcome	Sample size	SNP	*β*	se	*p*
Coffee intake	telomere length	ukb-b-5237	ieu-b-4879	472174	rs61928609	-0.02651	0.037897	0.484279
Coffee intake	telomere length	ukb-b-5237	ieu-b-4879	472174	rs62064918	-0.0253	0.037875	0.504156
Coffee intake	telomere length	ukb-b-5237	ieu-b-4879	472174	rs62121718	-0.02394	0.03789	0.527557
Coffee intake	telomere length	ukb-b-5237	ieu-b-4879	472174	rs630194	-0.0253	0.037951	0.504942
Coffee intake	telomere length	ukb-b-5237	ieu-b-4879	472174	rs641727	-0.02252	0.037895	0.552393
Coffee intake	telomere length	ukb-b-5237	ieu-b-4879	472174	rs6469262	-0.02453	0.037916	0.517718
Coffee intake	telomere length	ukb-b-5237	ieu-b-4879	472174	rs6544966	-0.02325	0.037891	0.539542
Coffee intake	telomere length	ukb-b-5237	ieu-b-4879	472174	rs6691876	-0.02088	0.037771	0.580463
Coffee intake	telomere length	ukb-b-5237	ieu-b-4879	472174	rs6937318	-0.01932	0.037538	0.606766
Coffee intake	telomere length	ukb-b-5237	ieu-b-4879	472174	rs6989976	-0.02388	0.037894	0.528494
Coffee intake	telomere length	ukb-b-5237	ieu-b-4879	472174	rs71429826	-0.02504	0.037837	0.508154
Coffee intake	telomere length	ukb-b-5237	ieu-b-4879	472174	rs723145	-0.02368	0.037899	0.532055
Coffee intake	telomere length	ukb-b-5237	ieu-b-4879	472174	rs730632	-0.02127	0.037774	0.57339
Coffee intake	telomere length	ukb-b-5237	ieu-b-4879	472174	rs73075167	-0.02036	0.03784	0.59049
Coffee intake	telomere length	ukb-b-5237	ieu-b-4879	472174	rs73123665	-0.02418	0.037882	0.52326

Exposure	Outcome	GWAS ID of exposure	GWAS ID of outcome	Sample size	SNP	*β*	se	*p*
Coffee intake	telomere length	ukb-b-5237	ieu-b-4879	472174	rs73228435	-0.02358	0.037908	0.533928
Coffee intake	telomere length	ukb-b-5237	ieu-b-4879	472174	rs75347775	-0.0235	0.037928	0.535536
Coffee intake	telomere length	ukb-b-5237	ieu-b-4879	472174	rs7585210	-0.02532	0.037814	0.503187
Coffee intake	telomere length	ukb-b-5237	ieu-b-4879	472174	rs763030	-0.02846	0.037401	0.446651
Coffee intake	telomere length	ukb-b-5237	ieu-b-4879	472174	rs76852278	-0.02273	0.037884	0.54858
Coffee intake	telomere length	ukb-b-5237	ieu-b-4879	472174	rs76881016	-0.02482	0.037878	0.512224
Coffee intake	telomere length	ukb-b-5237	ieu-b-4879	472174	rs77350503	-0.02224	0.037832	0.556646
Coffee intake	telomere length	ukb-b-5237	ieu-b-4879	472174	rs7743687	-0.02379	0.037899	0.530178
Coffee intake	telomere length	ukb-b-5237	ieu-b-4879	472174	rs780093	-0.01688	0.037671	0.654104
Coffee intake	telomere length	ukb-b-5237	ieu-b-4879	472174	rs7811609	-0.02648	0.037773	0.48324
Coffee intake	telomere length	ukb-b-5237	ieu-b-4879	472174	rs78267637	-0.02383	0.037939	0.529942
Coffee intake	telomere length	ukb-b-5237	ieu-b-4879	472174	rs78308709	-0.02461	0.037886	0.516016
Coffee intake	telomere length	ukb-b-5237	ieu-b-4879	472174	rs7989746	-0.02496	0.037842	0.50945
Coffee intake	telomere length	ukb-b-5237	ieu-b-4879	472174	rs8056750	-0.02552	0.037896	0.500737
Coffee intake	telomere length	ukb-b-5237	ieu-b-4879	472174	rs8068193	-0.02189	0.037808	0.562579

**Exposure**	**Outcome**	**GWAS ID of exposure**	**GWAS ID of outcome**	**Sample size**	**SNP**	***β***	**se**	***p***
Coffee intake	telomere length	ukb-b-5237	ieu-b-4879	472174	rs874573	-0.02185	0.037824	0.563511
Coffee intake	telomere length	ukb-b-5237	ieu-b-4879	472174	rs9296406	-0.02174	0.037805	0.565182
Coffee intake	telomere length	ukb-b-5237	ieu-b-4879	472174	rs9398171	-0.02228	0.037928	0.556956
Coffee intake	telomere length	ukb-b-5237	ieu-b-4879	472174	rs9650210	-0.02148	0.037793	0.569831
Coffee intake	telomere length	ukb-b-5237	ieu-b-4879	472174	rs9953004	-0.02524	0.037821	0.504519
Coffee intake	telomere length	ukb-b-5237	ieu-b-4879	472174	All	-0.02365	0.037622	0.529649

Supplementary Table S2. The leave-one-out analysis of filtered coffee intake on telomere length using IVW approach.

Exposure	Outcome	GWAS ID of exposure	GWAS ID of outcome	Sample size	SNP	*β*	se	*p*
Filtered coffee intake	telomere length	ukb-b-748	ieu-b-4879	472174	rs10468280	0.000534	0.018157	0.976518
Filtered coffee intake	telomere length	ukb-b-748	ieu-b-4879	472174	rs10875059	-0.00534	0.019084	0.779793
Filtered coffee intake	telomere length	ukb-b-748	ieu-b-4879	472174	rs10932789	-0.00661	0.018948	0.727048
Filtered coffee intake	telomere length	ukb-b-748	ieu-b-4879	472174	rs117372271	-0.00919	0.018375	0.617125
Filtered coffee intake	telomere length	ukb-b-748	ieu-b-4879	472174	rs1215980	-0.00637	0.019009	0.737387
Filtered coffee intake	telomere length	ukb-b-748	ieu-b-4879	472174	rs12535729	-0.00588	0.019115	0.758545
Filtered coffee intake	telomere length	ukb-b-748	ieu-b-4879	472174	rs144522705	-0.01133	0.017587	0.519355
Filtered coffee intake	telomere length	ukb-b-748	ieu-b-4879	472174	rs147912937	-0.00668	0.01889	0.723726
Filtered coffee intake	telomere length	ukb-b-748	ieu-b-4879	472174	rs16973000	-0.01392	0.016517	0.399197
Filtered coffee intake	telomere length	ukb-b-748	ieu-b-4879	472174	rs2470893	0.00598	0.017489	0.732404
Filtered coffee intake	telomere length	ukb-b-748	ieu-b-4879	472174	rs2830854	0.000524	0.018333	0.977194
Filtered coffee intake	telomere length	ukb-b-748	ieu-b-4879	472174	rs3004179	-0.00695	0.01917	0.716973
Filtered coffee intake	telomere length	ukb-b-748	ieu-b-4879	472174	rs4129316	0.000251	0.018053	0.988925
Filtered coffee intake	telomere length	ukb-b-748	ieu-b-4879	472174	rs6968865	-0.007	0.019494	0.719475

Filtered coffee intake	telomere length	ukb-b-748	ieu-b-4879	472174	rs74852036	-0.00341	0.018905	0.856649
Filtered coffee intake	telomere length	ukb-b-748	ieu-b-4879	472174	rs7610922	-0.00297	0.018865	0.875106
Filtered coffee intake	telomere length	ukb-b-748	ieu-b-4879	472174	rs76653299	-0.00547	0.019007	0.773388
Filtered coffee intake	telomere length	ukb-b-748	ieu-b-4879	472174	rs7788498	-0.00687	0.018897	0.716241
Filtered coffee intake	telomere length	ukb-b-748	ieu-b-4879	472174	rs78169764	-0.00215	0.018781	0.908925
Filtered coffee intake	telomere length	ukb-b-748	ieu-b-4879	472174	rs80353807	-0.0059	0.018994	0.756056
Filtered coffee intake	telomere length	ukb-b-748	ieu-b-4879	472174	rs9791176	-0.00623	0.018996	0.742992
Filtered coffee intake	telomere length	ukb-b-748	ieu-b-4879	472174	All	-0.00502	0.01817	0.782345

Supplementary Table S3. The leave-one-out analysis of instant coffee intake on telomere length using IVW approach.

Exposure	Outcome	GWAS ID of exposure	GWAS ID of outcome	Sample size	SNP	*β*	se	*p*
Instant coffee intake	telomere length	ukb-b-930	ieu-b-4879	472174	rs112601576	-0.02072	0.008541	0.015251
Instant coffee intake	telomere length	ukb-b-930	ieu-b-4879	472174	rs11649454	-0.01745	0.008543	0.041048
Instant coffee intake	telomere length	ukb-b-930	ieu-b-4879	472174	rs117671135	-0.01882	0.008605	0.02874
Instant coffee intake	telomere length	ukb-b-930	ieu-b-4879	472174	rs117947931	-0.01995	0.008617	0.020607
Instant coffee intake	telomere length	ukb-b-930	ieu-b-4879	472174	rs12510185	-0.021	0.008534	0.013855
Instant coffee intake	telomere length	ukb-b-930	ieu-b-4879	472174	rs143523613	-0.0204	0.008554	0.017072
Instant coffee intake	telomere length	ukb-b-930	ieu-b-4879	472174	rs144845508	-0.02183	0.008544	0.010616
Instant coffee intake	telomere length	ukb-b-930	ieu-b-4879	472174	rs148251241	-0.01811	0.008563	0.034405
Instant coffee intake	telomere length	ukb-b-930	ieu-b-4879	472174	rs1537362	-0.01999	0.008669	0.021133
Instant coffee intake	telomere length	ukb-b-930	ieu-b-4879	472174	rs167978	-0.02011	0.008595	0.019276
Instant coffee intake	telomere length	ukb-b-930	ieu-b-4879	472174	rs2171275	-0.02052	0.008555	0.016437
Instant coffee intake	telomere length	ukb-b-930	ieu-b-4879	472174	rs2472297	-0.01466	0.00865	0.090093
Instant coffee intake	telomere length	ukb-b-930	ieu-b-4879	472174	rs2599415	-0.02281	0.00854	0.007553
Instant coffee intake	telomere length	ukb-b-930	ieu-b-4879	472174	rs2726351	-0.01648	0.008577	0.054658

Instant coffee intake	telomere length	ukb-b-930	ieu-b-4879	472174	rs34667201	-0.01884	0.008606	0.028623
Instant coffee intake	telomere length	ukb-b-930	ieu-b-4879	472174	rs62418417	-0.01767	0.008555	0.038857
Instant coffee intake	telomere length	ukb-b-930	ieu-b-4879	472174	rs6968554	-0.02321	0.008737	0.007887
Instant coffee intake	telomere length	ukb-b-930	ieu-b-4879	472174	rs76197902	-0.019	0.00862	0.027519
Instant coffee intake	telomere length	ukb-b-930	ieu-b-4879	472174	rs78548721	-0.02043	0.00859	0.017374
Instant coffee intake	telomere length	ukb-b-930	ieu-b-4879	472174	rs80144686	-0.01845	0.008553	0.030996
Instant coffee intake	telomere length	ukb-b-930	ieu-b-4879	472174	All	-0.01952	0.008345	0.019309
